# Clinical Efficacy of Local Injection of Compound Betamethasone Versus Triamcinolone Acetonide in the Treatment of Hypertrophic Scars: A Retrospective Analysis of 126 Patients

**DOI:** 10.1111/jocd.70903

**Published:** 2026-05-10

**Authors:** Jiaqian Mao, Minghui He

**Affiliations:** ^1^ Department of Dermatologyt Ya'an Polytechnic College Affiliated Hospital Ya'an Sichuan China

**Keywords:** compound betamethasone, hypertrophic scar, intralesional corticosteroid injection, triamcinolone acetonide

## Abstract

**Background:**

Hypertrophic scars (HTS) are common complications of wound healing characterized by excessive fibroblast proliferation and collagen deposition. HTS causes local elevation, pruritus, and pain, significantly impacting aesthetics and quality of life. While triamcinolone acetonide (TA) and compound betamethasone (CB) are standard treatments, direct comparative studies regarding their efficacy and recurrence rates remain limited.

**Objective:**

To evaluate the clinical efficacy, safety, and recurrence rate over 6 months of local injection of TA and CB monotherapy in the treatment of HTS.

**Methods:**

We retrospectively analyzed 126 patients with HTS who received intralesional injection therapy at our hospital between December 2021 and December 2024. Patients were divided into the TA group (*n* = 63) and the CB group (*n* = 63) based on the injected drug. All patients were observed during treatment and followed for at least 6 months to monitor adverse reactions and recurrence. Scar assessment was performed using the Vancouver Scar Scale (VSS), combined with high‐frequency ultrasonography to measure scar thickness changes. Clinical efficacy was evaluated based on the percentage reduction in VSS scores.

**Results:**

Baseline characteristics were not significantly different between the two groups. Both CB and TA significantly reduced VSS scores and ultrasonographically measured scar thickness in patients with HTS; however, the CB group exhibited greater reductions in both VSS scores and scar thickness compared with the TA group. Evaluation of clinical efficacy revealed a total effective rate of 93.65% in the CB group versus 80.95% in the TA group. The incidence of adverse reactions was similar between the two groups, primarily consisting of local skin atrophy, pigmentation changes, and telangiectasia. The 6‐month recurrence rate was significantly lower for CB (3.17%) than for TA (15.87%, *p* = 0.015).

**Conclusion:**

Both CB and TA intralesional injections are effective in improving VSS scores, reducing scar thickness, and achieving high clinical efficacy rates in patients with HTS. However, CB demonstrates superior therapeutic outcomes and a significantly lower recurrence rate compared with TA, supporting its wider clinical application.

## 1 Introduction

As the largest organ of the human body, the skin forms a protective barrier between the body and the external environment. Injured skin typically heals through fibrotic scar tissue, which has permanent structural defects and impaired function, in order to maintain skin integrity [1]. Hypertrophic scars (HTS) are the primary pathological outcome following skin injury, characterized by abnormal proliferation of fibroblasts, excessive collagen deposition, and increased local scar volume and hardness [2, 3]. Approximately 15% of postoperative incisions and more than 70% of burn patients develop abnormal scar formation [4, 5]. HTS not only affects appearance but also causes pain, pruritus, hardening, and scar contracture, leading to social stigma, psychological trauma, and a reduced quality of life for patients [4]. Due to the high recurrence rate and prolonged treatment duration of HTS, there is currently no standardized treatment guideline or optimal therapeutic strategy.

HTS remains a major clinical challenge worldwide, and current therapeutic strategies show limited efficacy in achieving scarless healing or reversing fibrosis. Available treatments for hypertrophic scars include silicone gel, pressure therapy, intralesional corticosteroid injection, laser therapy, cryotherapy, surgical excision, and radiotherapy, with intralesional corticosteroid injection considered the first‐line and most commonly used approach [3, 6]. Triamcinolone Acetonide (TA) is widely used in clinical practice due to its potent anti‐inflammatory and anti‐fibrotic effects; however, local side effects such as dermal/subcutaneous atrophy, thinning, telangiectasia, and pigmentation changes are common. Moreover, multiple injections are often required to achieve satisfactory outcomes, with recurrence rates at 1 and 5 years reported at approximately 33% and 50%, respectively [7, 8]. On the other hand, clinical reports have indicated that Compound Betamethasone (CB)‐based injectable solutions are also widely used for scar treatment in China, with some experiences suggesting a milder effect and better tolerability. CB injection consists of betamethasone dipropionate and betamethasone sodium phosphate, combining rapid‐onset and long‐acting effects, and has demonstrated potential clinical advantages in improving scar hardness, structure, and associated symptoms [9]. Nevertheless, as a corticosteroid, CB may also cause steroid‐related adverse reactions [8]. Although intralesional corticosteroid injection is widely used for the treatment of HTS, direct comparative studies evaluating the efficacy, safety, and recurrence rates of CB and TA remain limited worldwide, and the available evidence is insufficient to determine their relative clinical advantages.

To address this gap and provide real‐world data, this study conducted a retrospective analysis of 126 patients with HTS, comparing local injection of CB and TA in terms of efficacy improvement, symptom relief, and incidence of adverse reactions, providing more reliable evidence for clinical practice.

## 2 Materials and Methods

### 2.1 Study Population

This study was a single‐center retrospective cohort study. Using a convenience sampling method, we included 126 patients who received treatment for HTS at our Hospital between December 2021 and December 2024. Patients were divided into the CB group (*n* = 63) and the TA group (*n* = 63) based on the clinical pharmacological intervention received. Treatment regimens were determined by the attending physicians based on clinical experience, scar characteristics, and patient preference. No randomization was performed. The severity of HTS was evaluated using the Vancouver Scar Scale (VSS), which assesses vascularity, pigmentation, pliability, and height. Baseline VSS scores showed no significant differences between the CB and TA groups, establishing comparability between the cohorts. Hypertrophic scars were diagnosed by qualified dermatologists based on clinical presentation, ensuring a strict differentiation between HTS and keloids. HTS was defined as fibroproliferative lesions confined within the boundaries of the original injury, whereas keloids were defined as abnormal growths extending beyond the initial wound borders. Only patients with HTS were included. The study protocol was approved by the Medical Ethics Committee of Ya'an Polytechnic College Affiliated Hospital (Approval No.: IRB‐PJ‐KY/2025‐10). This study was a single‐center retrospective clinical analysis; all data were obtained from the electronic medical record system and anonymized, complying with the exemption clause for informed consent under the Declaration of Helsinki. Therefore, informed consent was waived.

Inclusion criteria: (1) Age ≥ 18 years; (2) Clinical diagnosis of hypertrophic scars (non‐keloid), with scar formation ≥ 3 months; (3) No other local interventions (e.g., laser therapy, surgery, radiotherapy) in the 3 months prior to intralesional injection; (4) Received monotherapy with either CB or TA intralesional injection and completed a full treatment course (3–5 sessions); (5) Follow‐up period ≥ 6 months, with complete clinical data and VSS evaluation records.

Exclusion criteria: (1) Diagnosis of keloid or presence of mixed‐type scars; (2) Severe cardiac, hepatic, renal insufficiency, or other major organ diseases; (3) Concurrent skin infection, dermatologic disease (e.g., psoriasis, scleroderma), or open wound at the injection site; (4) Pregnant or lactating women; (5) History of allergy or adverse reaction to corticosteroid therapy; or (6) Incomplete records or loss to follow‐up.

### 2.2 Treatment Methods

Intralesional injections were administered using a WZ‐02 needle‐free injector. In the CB group, patients received CB injection (Huapont Pharmaceutical, Chongqing, China, Drug Approval No.: H20093412; Specification: 1 mL contains 5 mg betamethasone dipropionate and 2 mg betamethasone sodium phosphate) at 1 mL per session. Patients in the TA group received TA injection (Jida Pharmaceutical, Kunming, China, Drug Approval No.: H53021604; Specification: 1 mL contains 40 mg) at 1 mL per session. Both agents were mixed in a 1:1 ratio with 1% Lidocaine (Guorui Pharmaceutical, Sichuan, China, Drug Approval No.: H20055048), with a total injection volume of 1 mL per session. All procedures were performed by experienced dermatologists following a standardized protocol. Prior to injection, the hypertrophic scar area was disinfected with 0.5% povidone‐iodine. The solution was then injected slowly and horizontally into the scar tissue, ensuring the medication remained confined to the lesion and avoiding infiltration into the surrounding or underlying normal tissue. Injection endpoint was indicated by slight blanching of the scar surface. The number of injection points was adjusted according to the scar surface area to ensure uniform drug distribution and therapeutic consistency. Following the procedure, pressure was applied with sterile cotton swabs to achieve hemostasis. Strict aseptic techniques were maintained to prevent local infection. Injections were performed once every 4 weeks, with 3–5 sessions administered based on clinical condition.

### 2.3 VSS for Scar Assessment

Scar assessment was performed before treatment and at 1 and 3 months after the final treatment using the VSS [10], which evaluates four dimensions: vascularity (0–5 points), pigmentation (0–2 points), pliability (0–5 points), and scar thickness (0–3 points). The total VSS score is the sum of all dimensions, with higher scores indicating more severe scar proliferation.

### 2.4 Clinical Efficacy Evaluation

Clinical efficacy was determined based on the percentage reduction in VSS scores from baseline [11]. The efficacy index was calculated as follows: Efficacy Index (%) = (VSS score before treatment − VSS score after treatment)/VSS score before treatment × 100%.

Cured: Efficacy index ≥ 90%; Markedly effective: 60% ≤ Efficacy index < 90%; Effective: 20% ≤ Efficacy index < 60%; Ineffective: Efficacy index < 20%.

$$\text{Total effective rate}=\left(\text{number of cured}+\text{markedly effective}+\text{effective patients}\right)/\text{total patients}\times 100\%.$$

### 2.5 Ultrasonographic Measurement of Scar Thickness

High‐frequency ultrasonography (10–15 MHz) was used to measure scar thickness at the same anatomical site perpendicular to the scar tissue. Three consecutive measurements were averaged to obtain the final thickness. Measurements were recorded before treatment and 3 months after treatment. All measurements were performed by the same sonographer to minimize inter‐observer variability.

### 2.6 Adverse Reactions and Recurrence

Local adverse reactions occurring during treatment and within 6 months post‐treatment were recorded. These excluded injection‐related pain or bleeding and primarily included corticosteroid‐induced reactions such as infection, skin atrophy, pigmentation changes, acne, and telangiectasia. Recurrence was defined as the reappearance of elevation and induration at the original scar site, accompanied by symptoms such as erythema or pruritus. Recurrence at 6 months was assessed via telephone or outpatient follow‐up, defined as re‐elevation and hardening of the original lesion, accompanied by significant pain, pruritus, and erythema. No patients were lost to follow‐up.

### 2.7 Statistical Analysis

Data were analyzed using SPSS 27.0 statistical software. Continuous variables were tested for normality. Normally distributed data are expressed as mean ± standard deviation ($\bar{x}\pm s$); intergroup comparisons were conducted using the independent‐samples *t‐*test, while intragroup comparisons (pre‐ vs. post‐treatment) were analyzed using the paired samples *t*‐test. Non‐normally distributed data are expressed as median (interquartile range, IQR) [M(P25, P75)] and were analyzed using the Mann–Whitney *U* test for intergroup comparisons and the Wilcoxon signed‐rank test for intragroup comparisons. Categorical data are expressed as number of cases (percentage) [*n* (%)] and compared using the chi‐square (*χ* ^2^) test. A two‐sided *p*‐value < 0.05 was considered statistically significant.

## 3 Results

### 3.1 Comparison of Baseline Characteristics

A total of 126 patients with HTS were included in this study, with 63 patients in the TA group and 63 in the CB group. There were no significant differences between the two groups in terms of age, sex, BMI, disease duration, treatment history, total number of treatments, scar location, pre‐treatment VSS scores, or ultrasonographic scar thickness (*p* > 0.05; Table 1), indicating that baseline characteristics were balanced and comparable.

**TABLE 1:** Comparison of baseline characteristics between the two groups.

Baseline characteristics	TA group (*n* = 63)	CB group (*n* = 63)	Effect size (95% CI)	*χ* ^2^/*Z*/*t*	*p*
Age (years)	39 (31, 46)	41 (31, 48)	−0.088 [−0.284, 0.105]	−0.85	0.396
Sex			1.466 [0.727, 2.956]	1.143	0.285
Male	35 (44.44)	29 (46.03)	—		
Female	28 (56.56)	34 (54.97)	—		
BMI (kg/m^2^)	22.36 ± 2.86	23.13 ± 2.72	−0.276 [−0.631, 0.078]	−1.552	0.123
Disease duration (months)	7.92 (3.33, 13.18)	8.89 (4.1, 13.88)	−0.024 [−0.232, 0.183]	−0.234	0.815
Treatment history	13 (20.63)	10 (15.87)	1.378 [0.554, 3.425]	0.479	0.489
Total number of treatments	5 (4, 5)	5 (5, 5)	−0.046 [−0.215, 0.098]	−0.58	0.562
Scar location			0.052 [0.000, 0.183]	0.347	0.841
Head and neck	15 (23.81)	13 (20.63)	—	—	—
Trunk	23 (36.51)	26 (41.27)	—	—	—
Extremities	25 (39.68)	24 (38.10)	—	—	—
Pre‐treatment VSS scores	8 (7, 9)	8 (7, 9)	−0.02 [−0.213, 0.177]	−0.193	0.847
Pre‐treatment ultrasonographic scar thickness (mm)	5.87 ± 0.79	5.86 ± 0.78	0.012 [−0.34, 0.365]	0.069	0.945

*Note:* Data are presented as median (interquartile range, Q1–Q3), mean ± standard deviation, or frequency (percentage) [*n* (%)].

Abbreviations: BMI, body mass index; CB, compound betamethasone; CI, confidence interval; TA, triamcinolone acetonide; VSS, Vancouver Scar Scale.

### 3.2 Comparison of VSS Scores Before and After Intralesional Injection

**FIGURE 1:**
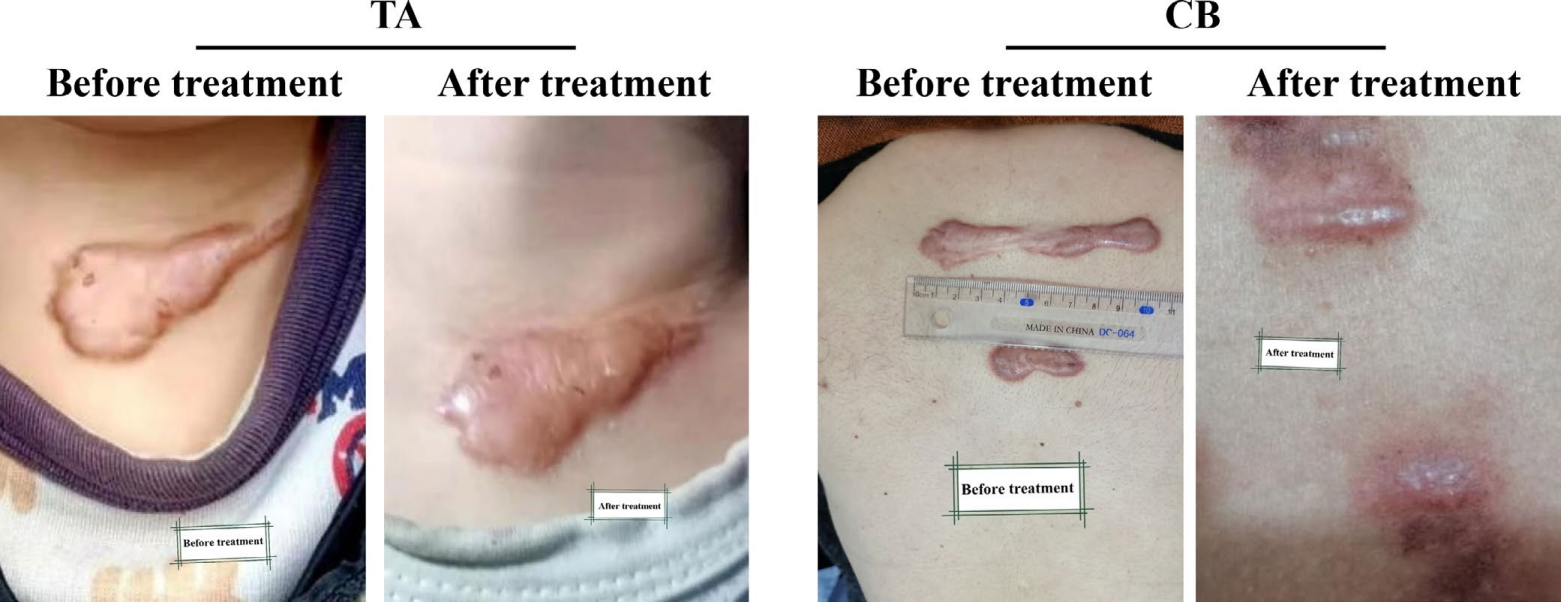
Representative clinical photographs of HTS before and after intralesional injection treatment with CB and TA. CB, compound betamethasone; TA, triamcinolone acetonide.

To visually demonstrate the therapeutic outcomes, representative clinical photographs from both groups were selected. As shown in Figure 1, both groups exhibited lightened scar pigmentation and increased pliability following three treatment cycles. Notably, the degree of improvement in the CB group was more pronounced than that observed in the TA group.

Pre‐treatment VSS scores did not differ significantly between the two groups (*p* = 0.847). Both groups showed a significant reduction in VSS scores after treatment compared with baseline (*p* < 0.001). Between‐group comparisons demonstrated statistically significant differences at 1 month (*Z* = −2.442, *p* = 0.015) and 3 months (*Z* = −3.781, *p* < 0.001) post‐treatment. Additionally, the change in VSS at 3 months (ΔVSS) differed significantly between groups (*Z* = −4.558, *p* < 0.001) (Table 2), indicating that CB produced a greater improvement in VSS scores compared with TA.

**TABLE 2:** Comparison of VSS scores and score changes before and after CB and TA treatment.

Characteristics	TA group (*n* = 63)	CB group (*n* = 63)	Effect size (95% CI)	*Z*	*p*
Pre‐treatment VSS scores	8 (7, 9)	8 (7, 9)	−0.02 [−0.213, 0.177]	−0.193	0.847
VSS score at 1 month post‐treatment	7 (5, 8)	6 (5, 7)	0.247 [0.057, 0.441]	−2.442	**0.015**
VSS score at 3 months post‐treatment	5 (4, 6)	4 (3, 5)	0.382 [0.201, 0.557]	−3.781	**< 0.001**
ΔVSS	3 (2, 4)	4 (3, 5)	−0.461 [−0.635, −0.295]	−4.558	**< 0.001**
Effect size (95% CI)	0.7281 [0.5822, 0.8495]	0.9101 [0.8155, 0.9801]			
*Z*	−6.940	−6.950		—	—
*p*	**< 0.001**	**< 0.001**		—	—

*Note:* Data are presented as the median (interquartile range, Q1–Q3). Bold values indicate statistically significant differences at the *p* < 0.05 level.

Abbreviations: CB, compound betamethasone; CI, confidence interval; TA, triamcinolone acetonide; VSS, Vancouver Scar Scale.

### 3.3 Comparison of Ultrasonographic Scar Thickness Before and After Injection

Ultrasonographic scar thickness before and after treatment is shown in Table 3. Pre‐treatment thickness did not differ significantly between groups (*p* = 0.945), confirming baseline consistency. Post‐treatment, scar thickness decreased significantly in both groups (*p* < 0.001). In the TA group, thickness decreased from 5.87 ± 0.79 mm to 4.22 ± 0.92 mm (*t* = 10.798, *p* < 0.001), whereas in the CB group, thickness decreased from 5.86 ± 0.78 mm to 3.68 ± 0.70 mm (*t* = 16.508, *p* < 0.001). Post‐treatment thickness also differed significantly between groups (*t* = 3.748, *p* < 0.001), with CB showing a greater reduction than TA (*p* < 0.001). These results suggest that CB is more effective than TA in reducing scar thickness.

**TABLE 3:** Comparison of ultrasonographic scar thickness before and after CB and TA treatment.

Ultrasonographic scar thickness (mm)	TA group (*n* = 63)	CB group (*n* = 63)	Effect size (95% CI)	*t*	*p*
Before treatment	5.87 ± 0.79	5.86 ± 0.78	0.012 [−0.34, 0.365]	0.069	0.945
At 3 months post‐treatment	4.22 ± 0.92	3.68 ± 0.7	0.668 [0.305, 1.03]	3.748	**< 0.001**
Δ Scar thickness	1.65 ± 0.47	2.18 ± 0.58	−1.019 [−1.394, −0.644]	−5.718	**< 0.001**
Effect size (95% CI)	3.5065 [2.8286, 4.1845]	3.7861 [3.0613, 4.5109]			
*t*	27.832	30.051		—	—
*p*	**< 0.001**	**< 0.001**		—	—

*Note:* Data are presented as mean ± standard deviation (Mean ± SD). Bold values indicate statistically significant differences at the *p* < 0.05 level.

Abbreviations: CB, compound betamethasone; CI, confidence interval; TA, triamcinolone acetonide.

### 3.4 Comparison of VSS Score Improvement and Ultrasonographic Scar Thickness Reduction by Anatomical Location

A stratified analysis was performed to evaluate the therapeutic efficacy of TA and CB across different anatomical sites. The results indicated that for both ΔVSS scores and Δscar thickness, CB yielded significantly greater improvements across the head and neck, trunk, and extremities compared to the TA group (*p* < 0.05, Table 4).

**TABLE 4:** Comparison of scar improvement between groups by anatomical location (ΔVSS and ΔScar thickness).

Characteristics	Scar location	TA group (*n* = 63)	CB group (*n* = 63)	Effect size (95% CI)	*Z*/*t*	*p*
ΔVSS	Head and neck	3 ± 1.07	4 ± 1.22	−0.860 [−1.642, −0.078]	−2.308	**0.029**
	Trunk	3 (2, 4)	5 (3, 5)	−0.4557 [−0.7062, −0.1843]	−2.899	**0.004**
	Extremities	3 (2, 4)	4 (4, 5)	−0.4336 [−0.6770, −0.1216]	−2.74	**0.006**
ΔScar thickness	Head and neck	1.57 ± 0.50	2.30 ± 0.48	−1.600 [−2.455, −0.745]	−3.950	**< 0.001**
	Trunk	1.63 ± 0.44	2.11 ± 0.51	−1.0751 [−1.6989, −0.4513]	−3.542	**< 0.001**
	Extremities	1.71 ± 0.49	2.19 ± 0.69	−0.7971 [−1.3888, −0.2053]	−2.802	**0.008**

*Note:* Data are presented as median (interquartile range, Q1–Q3) or mean ± standard deviation. Bold values indicate statistically significant differences at the *p* < 0.05 level.

Abbreviations: CB, compound betamethasone; CI, confidence interval; TA, triamcinolone acetonide; VSS, Vancouver Scar Scale.

### 3.5 Clinical Efficacy Analysis

Clinical efficacy was evaluated in all 126 HTS patients who received intralesional TA or CB. The total effective rate in the CB group was 93.65%, compared with 80.95% in the TA group. CB demonstrated significantly higher clinical efficacy than TA (*p* = 0.032) (Table 5), indicating superior overall treatment effectiveness.

**TABLE 5:** Comparison of clinical efficacy of intralesional TA or CB injection, *n* (%).

Cases	TA group (*n* = 63)	CB group (*n* = 63)	Effect size (95% CI)	*χ* ^2^	*p*
Clinical efficacy	51 (80.95%)	59 (93.65%)	0.264 [0.082, 0.849]	4.582	**0.032**
Cured	0 (0%)	1 (1.59%)			
Markedly effective	6 (9.52%)	14 (22.22%)			
Effective	45 (71.43%)	44 (69.84%)			
Ineffective	12 (19.05%)	4 (6.35%)			

*Note:* Data are presented as frequency (percentage) [*n* (%)]. Bold values indicate statistically significant differences at the *p* < 0.05 level.

Abbreviations: CB, compound betamethasone; CI, confidence interval; TA, triamcinolone acetonide.

### 3.6 Adverse Reactions and Recurrence

During treatment and within 6 months post‐treatment, both groups experienced varying degrees of local adverse reactions, including skin atrophy, pigmentation changes, telangiectasia, and infection. Adverse reactions occurred in 13 patients (20.63%) in the TA group and 11 patients (17.46%) in the CB group, with no significant difference between groups (*χ* ^2^ = 0.206, *p* = 0.65) (Table 5). At 6‐month follow‐up, recurrence was observed in 2 patients (3.17%) in the CB group and 10 patients (15.87%) in the TA group, representing a statistically significant difference (*χ* ^2^ = 5.895, *p* = 0.015) (Table 6). In summary, while the incidence of adverse reactions was comparable between TA and CB, CB demonstrated a significantly lower 6‐month recurrence rate.

**TABLE 6:** Adverse reactions and recurrence rates after intralesional TA or CB injection.

Cases	TA group (*n* = 63)	CB group (*n* = 63)	Effect size (95% CI)	*χ* ^2^	*p*
Adverse reactions	13 (20.63%)	11 (17.46%)	1.227 [0.522, 2.885]	0.206	0.65
Skin atrophy	3 (4.76%)	2 (3.17%)			
Pigmentation changes	6 (9.52%)	5 (7.94%)			
Localized erythema	1 (1.59%)	1 (1.59%)			
Telangiectasia	2 (3.17%)	3 (4.76%)			
Infection	1 (1.59%)	0 (0%)			
Recurrence rate	10 (15.87%)	2 (3.17%)	5.679 [1.222, 26.439]	5.895	**0.015**

*Note:* Data are presented as frequency (percentage) [*n* (%)]. Bold values indicate statistically significant differences at the *p* < 0.05 level.

Abbreviations: CB, compound betamethasone; CI, confidence interval; TA, triamcinolone acetonide.

## 4 Discussion

Corticosteroids exert anti‐scar effects through multiple mechanisms, including anti‐inflammatory activity, inhibition of fibroblast proliferation and collagen synthesis, and suppression of angiogenesis and fibroblast activity. The core mechanism involves modulating the expression of inflammation‐related genes, thereby reducing abnormal tissue repair responses [12, 13]. Compared to systemic administration, intralesional injection achieves higher drug concentrations within the lesion and is thus widely utilized in the clinical treatment of pathological scars. Previous studies have shown that intralesional corticosteroid injection can induce regression of pathological scars more rapidly than oral medications [14, 15, 16]. However, variations in potency, onset of action, and local retention time among different formulations may lead to divergent efficacy and safety profiles across different scar types and individuals. Furthermore, factors such as injection dose, interval, and technique significantly influence therapeutic outcomes, contributing to the heterogeneity observed in current research findings [9, 17].

The results of this study demonstrated that intralesional injection of both CB and TA significantly improved VSS scores and scar thickness, which is generally consistent with previous research on corticosteroid therapy for scars [13, 18, 19]. This consistency may stem from shared pathophysiological mechanisms among different types of pathological scars (e.g., hypertrophic scars, keloids, and acne scars), such as abnormal fibroblast proliferation, excessive collagen deposition, and chronic inflammation. Consequently, corticosteroids exert therapeutic effects across various scar types by inhibiting inflammatory responses and collagen synthesis. Nonetheless, differences in biological behavior, invasiveness, and recurrence tendency among scar types may account for the variations in efficacy reported across studies.

A further comparison of the two agents revealed that the CB group outperformed the TA group in terms of ΔVSS scores, improvement in scar thickness, and overall effective rate, suggesting that CB offers distinct advantages in improving scar appearance. This discrepancy in efficacy may be attributed to differences in pharmacokinetic characteristics and corticosteroid potency. The superior efficacy of CB likely results from its unique formulation, which consists of fast‐acting betamethasone sodium phosphate and long‐acting betamethasone dipropionate. The former is highly water‐soluble and rapidly absorbed for a quick onset of action, while the latter is highly lipid‐soluble and absorbed slowly, providing a sustained effect. CB is the only injectable corticosteroid that combines both rapid‐onset and sustained‐release components [20]. Additionally, the potency of betamethasone is five times that of the intermediate‐acting corticosteroid triamcinolone acetonide. Its “rapid + long‐acting” pharmacokinetic profile may enable more persistent inhibition of inflammatory responses, fibroblast hyperplasia, and collagen synthesis [21], partially explaining the superior performance of CB in reducing scar thickness and VSS scores in this study. Existing research has explored the mechanisms of TA in scar management in depth. For instance, Diegelmann et al. found that abnormal scar formation is closely linked to disordered collagen metabolism; TA may promote collagen degradation by regulating the balance between collagenase and its inhibitors, thereby reducing excessive collagen deposition [22]. Furthermore, TA has been shown to inhibit the expression of COL1, COL3, and α‐SMA, as well as the proliferation, migration, and invasion of human fibroblasts, significantly improving cutaneous scars [23]. In contrast, molecular research on CB in scar treatment remains relatively limited. Based on its pharmacokinetic properties, we hypothesize that CB may achieve superior outcomes by providing more sustained inhibition of fibroblast activity and collagen deposition, though this hypothesis requires further validation through basic research.

Regarding safety, both drugs produced common corticosteroid‐related adverse reactions, including skin atrophy, pigmentation changes, and telangiectasia [24], but the incidence was similar between groups. This suggests that localized corticosteroid‐related adverse events are more closely related to injection dose, frequency, and local tissue response rather than the specific type of drug. In this study, the number of treatments and overall therapeutic exposure were similar between groups, which likely accounts for the lack of significant difference in adverse reaction rates.

Notably, follow‐up data revealed that the recurrence rate in the TA group was significantly higher than in the CB group. This difference may be attributed to the long‐acting component of CB, which maintains stable local drug concentrations, thereby achieving continuous inhibition of fibroblast proliferation and collagen deposition and reducing rebound scar growth after treatment cessation. This finding suggests that CB may have a significant clinical advantage in reducing HTS recurrence.

While both CB and TA intralesional injections effectively improved VSS scores and scar thickness in HTS patients, CB demonstrated advantages in improving scar appearance and lowering recurrence rates. However, several limitations must be acknowledged. First, as a single‐center retrospective study where group assignment was based on clinical practice, selection and information biases are inevitable, and consistency in treatment cycles across all patients cannot be fully guaranteed. To mitigate the impact of variations in therapeutic exposure, we restricted the number of treatments to 3–5 sessions. The lack of statistical difference in total treatment sessions between groups suggests comparable therapeutic intensity. Second, the sample size was relatively limited, and the 6‐month follow‐up period was insufficient to evaluate long‐term recurrence. Third, although adverse reactions were recorded, we did not perform a systematic stratified analysis of their correlation with specific injection protocols. Fourth, the evaluation relied on clinical scales and imaging without histological or molecular biological confirmation, leaving the specific biological mechanisms of the efficacy gap unelucidated. In future research, we intend to conduct prospective, randomized controlled trials with strictly standardized injection protocols. By incorporating long‐term follow‐up alongside imaging and molecular biomarker assessments, we aim to evaluate the impact of various corticosteroid regimens and administration techniques on long‐term clinical outcomes, recurrence rates, and adverse effects. Such studies will serve to further validate our findings and provide a deeper exploration of the underlying biological mechanisms.

Overall, both CB and TA intralesional injections effectively improve the clinical manifestations of HTS, with CB showing superior outcomes in scar appearance and recurrence reduction. Given the retrospective nature of this study, further high‐quality research is required to validate its clinical value.

## Author Contributions

Jiaqian Mao: contributed to conceptualization, data curation, formal analysis, investigation, methodology, project administration, resources, software, supervision, validation, visualization, and writing – original draft. Minghui He: contributed to conceptualization, methodology, supervision, project administration, resources, and writing – review and editing. All authors have read and approved the final version of the manuscript.

## Funding

The authors have nothing to report.

## Ethics Statement

This study was approved by the Ethics Committee of Ya'an Polytechnic College Affiliated Hospital (Approval No.: IRB‐PJ‐KY/2025‐10). This was a single‐center retrospective clinical study. All data were obtained from the electronic medical record system and were anonymized. In accordance with the Declaration of Helsinki, the requirement for informed consent was waived.

## Consent

Written informed consent was obtained from the patients for publication of their clinical information and accompanying images.

## Conflicts of Interest

The authors declare no conflicts of interest.

## Data Availability

The data used and/or analyzed during the current study are available from the corresponding author.
